# Endovanilloids are potential activators of the trigeminovascular nocisensor complex

**DOI:** 10.1186/s10194-016-0644-7

**Published:** 2016-05-17

**Authors:** Mária Dux, Éva Deák, Noémi Tassi, Péter Sántha, Gábor Jancsó

**Affiliations:** Department of Physiology, University of Szeged, Dóm tér 10, H-6720 Szeged, Hungary

**Keywords:** Dura mater encephali, Endovanilloid, Meningeal blood flow, Trigeminovascular nocisensor complex, Headache, Transient receptor potential vanilloid 1

## Abstract

**Background:**

In the dura mater encephali a significant population of trigeminal afferents coexpress the nociceptive ion channel transient receptor potential vanilloid type 1 (TRPV1) receptor and calcitonin gene-related peptide (CGRP). Release of CGRP serves the central transmission of sensory information, initiates local tissue reactions and may also sensitize the nociceptive pathway. To reveal the possible activation of meningeal TRPV1 receptors by endogenously synthetized agonists, the effects of arachidonylethanolamide (anandamide) and N-arachidonoyl-dopamine (NADA) were studied on dural vascular reactions and meningeal CGRP release.

**Methods:**

Changes in meningeal blood flow were measured with laser Doppler flowmetry in a rat open cranial window preparation following local dural applications of anandamide and NADA. The release of CGRP evoked by endovanilloids was measured with ELISA in an in vitro dura mater preparation.

**Results:**

Topical application of NADA induced a significant dose-dependent increase in meningeal blood flow that was markedly inhibited by pretreatments with the TRPV1 antagonist capsazepine, the CGRP antagonist CGRP_8–37_, or by prior systemic capsaicin desensitization. Administration of anandamide resulted in minor increases in meningeal blood flow that was turned into vasoconstriction at the higher concentration. In the in vitro dura mater preparation NADA evoked a significant increase in CGRP release. Cannabinoid CB1 receptors of CGRP releasing nerve fibers seem to counteract the TRPV1 agonistic effect of anandamide in a dose-dependent fashion, a result which is confirmed by the facilitating effect of CB1 receptor inhibition on CGRP release and its reversing effect on the blood flow.

**Conclusions:**

The present findings demonstrate that endovanilloids are potential activators of meningeal TRPV1 receptors and, consequently the trigeminovascular nocisensor complex that may play a significant role in the pathophysiology of headaches. The results also suggest that prejunctional CB1 receptors may modulate meningeal vascular responses.

## Background

The pathophysiology of primary headaches is thought to involve an activation of trigeminal sensory nerves that densely innervate the dura mater encephali. The activation of trigeminal peptidergic dural afferents elicits both nociceptive and local vascular responses. Prolonged activation of meningeal primary afferents may even sensitize second order neurons of the nociceptive pathway in the caudal trigeminal nucleus [1, 2]. Neurogenic inflammation of meningeal tissues is a process that accompanies nociception and the generation of pain and is therefore frequently used as a readout of nociceptive events possibly leading to headaches [3]. A great body of experimental evidence indicates that in a variety of organs neurogenic inflammatory responses are mediated by chemosensitive primary sensory neurons which can be selectively activated with capsaicin. Importantly, chemosensitive afferents besides conveying nociceptive information to the central nervous system release vasoactive peptides, such as calcitonin gene-related peptide (CGRP) and substance P (SP) from their peripheral endings producing a sterile neurogenic inflammation [4–8]. Recent studies in our laboratory have revealed that a significant population of meningeal sensory nerves is sensitive to capsaicin and expresses the transient receptor potential vanilloid type 1 (TRPV1) receptor [9–11], a non-selective cation channel that serves as a molecular integrator of different noxious stimuli, such as heat, low pH and chemical irritants like capsaicin [12, 13]. Accordingly, stimulation of dural sensory nerves with capsaicin results in an increase in meningeal blood flow which is mediated by CGRP via the activation of the TRPV1 receptor [9, 14]. In the meninges, the release of neuropeptides may be accompanied by an increase in vascular permeability and degranulation of mast cells besides arterial vasodilatation [15].

Based on experimental and clinical observations a pathophysiological role for meningeal TRPV1 activation in primary headaches and the TRPV1 receptor as a novel target for antimigraine drugs have been suggested. Hence, the specific antimigraine drug sumatriptan has been shown to inhibit the TRPV1-mediated activation of trigeminal ganglion neurons innervating the rat dura mater [16]. The use of kinase inhibitors counteracting an enhanced activity or an increased expression of the TRPV1 receptor was also suggested as a promising new approach of headache therapy [17]. Desensitization of the receptor by the TRPV1 agonist olvanil modulated neuronal activity within the trigeminocervical complex by acting on both vanilloid and cannabinoid receptors [18].

Activation of meningeal chemosensitive afferents expressing TRPV1 nociceptive channels may initiate a complex interplay among the different components of the trigeminovascular nocisensor complex that consists of the trigeminovascular chemosensitive primary afferent neurons with their peripheral and central processes, the meningeal vascular bed and dural mast cells and macrophages [19]. The events, which follow the activation of dural chemosensitive nocisensors, may be regarded as components of a positive feedback regulation, which may augment the initial vascular and nociceptive responses. In addition, neurogenic sensory vasodilatation may have also beneficial effects by removing tissue metabolites inducing, aggravating or maintaining headache attacks [19].

Clinical studies support the role of CGRP in the pathophysiology of headaches; during migraine attacks increased levels of CGRP could be measured in jugular venous blood collected from the affected side [20]. Further, CGRP receptor antagonists seem to be promising new drugs in the medication of migraine attacks since they reduce significantly the severity of headache pain [21, 22].

The significance of chemosensitive primary sensory neurons in headache mechanisms is further supported by the observation that some pathophysiological events may sensitize meningeal sensory nerves [11]. Further, the hypothesis has also been put forward that peripheral sensitization may be responsible for the intracranial hypersensitivity observed in migraine and other types of headaches when otherwise innocuous stimuli or even pulsatile changes in meningeal blood flow or intracranial pressure activate the trigeminal nociceptive pathway [23]. Prolonged activation of the primary sensory neurons may also alter the excitability of second-order neurons in the caudal trigeminal nuclear complex leading to the central sensitization of the nociceptive pathway which, in turn, increases the transmission of nociceptive information [24].

Different metabolites of membrane lipids have been recently characterized as endogenous activators of the TRPV1 receptor [25]. Many endovanilloids were shown to act also as endogenous cannabinoids [26]. Arachidonylethanolamide (anandamide) is probably the most widely studied endogenous ligand in the trigeminal system that acts on both cannabinoid (CB) and TRPV1 receptors. During migraine attacks endogenous ligands of the TRPV1 receptor may play more important role in the activation of peptidergic nociceptive primary afferents than heat or low pH. Putative endovanilloids may mediate meningeal vascular reactions in a similar way as capsaicin, through the release of CGRP induced via the activation of the TRPV1 receptor. In studying the biological activity of endovanilloid compounds, simultaneous activations of the TRPV1 and the CB receptors with pre- and postsynaptic localizations should be considered. In the trigeminal system cannabinoid CB1 receptor immunoreactive neurons were found mainly in the maxillary and mandibular divisions of the trigeminal nerve [27]. These branches of the trigeminal nerve innervate the temporal part of the dura mater where its main arterial blood vessels, the branches of the middle meningeal artery are also localized. Earlier observations indicate, that activation of trigeminal CB1 receptors inhibited dural vasodilatation brought about by electrical stimulation of the dura mater [28], and the release of CGRP induced by thermal stimulation in an in vitro dura mater preparation [29].

The aim of this study was to reveal the contribution of intracranial TRPV1 and CB1 receptor activation to changes in meningeal blood flow elicited by topically applied endogenous vanilloid/cannabinoid compounds, anandamide and N-arachidonoyl-dopamine (NADA), which have been previously identified in dorsal root ganglion neurons [30–33].

## Methods

### Experimental animals and surgery

Experiments were approved by the Ethical Committee for Animal Care of the University of Szeged (XIV/00065/2011). Study procedures were carried out in accordance with the Directive 2010/63/EU of the European Parliament. All efforts were made to minimize the number of animals used and their suffering. Control and capsaicin-desensitized adult male Wistar rats weighing 300–350 g were used, the number of animals in different experimental groups were between n: 6–13. Animals were raised and maintained under standard laboratory conditions. Capsaicin desensitization of animals was induced by subcutaneous injections of capsaicin on three consecutive days at increasing doses of 10, 20 and 100 mg/kg [34]. Intact animals and rats given the solvent for capsaicin (6 % ethanol and 8 % Tween 80 in saline) served as controls. Rats were anaesthetized with an initial dose of thiopental sodium (120 mg/kg, i.p. Thiopental, Biochemie GmbH, Austria). Additional doses of thiopental sodium (25 mg/kg i.p.) were administered throughout the experiment to avoid changes of systemic blood pressure or nociceptive reactions to noxious stimuli. Systemic blood pressure was recorded with a pressure transducer via a cannula inserted into the femoral artery. The animals were tracheotomized and breathed spontaneously [9]. The body temperature was kept at 37–37.5 °C with a heating pad. A cranial window for the measurement of dural blood flow was prepared according to Kurosawa et al. [35]; the head of the animal was fixed in a stereotaxic frame, the scalp was removed and the parietal bone was exposed on one side. A cranial window was drilled with a saline-cooled drill into the exposed parietal bone.

### Drug application

The open cranial window was filled with a modified synthetic interstitial fluid (SIF) containing (in mM) 135 NaCl, 5 KCl, 1 MgCl_2_, 5 CaCl_2_, 10 glucose and 10 Hepes [36]. Stock solutions of capsaicin, capsazepine, anandamide, NADA and the CB1 receptor antagonist AM 251 were prepared. Capsaicin (32 mM) and capsazepine (1 mM) were dissolved in saline containing 6 % ethanol and 8 % Tween 80, stock solutions of anandamide (14 mM), NADA (11 mM) and AM 251 (10 mM) were prepared with ethanol. Before the experiment drugs were further diluted with SIF to their final concentration.

At the beginning of the experiments the blood flow increasing effect of capsaicin (100 nM) was measured, then anandamide or NADA were applied at increasing concentrations (anandamide 100 nM, 1 μM and 10 μM, NADA 10 nM, 100 nM and 1 μM). To minimize the desensitizing effect of repeated vanilloid applications, in this series of experiments drugs were applied for 3 min.

To determine the contribution of TRPV1-receptors and the role of CGRP in the endovanilloid-induced changes in meningeal blood flow, the TRPV1-receptor antagonist capsazepine (10 μM) or the CGRP-receptor antagonist CGRP_8–37_ (100 μM) were applied onto the exposed surface of the dura mater, respectively. Five min later, NADA (100 nM) was administered for 3 min. In capsaicin-desensitized animals meningeal blood flow changes induced by the topical application of capsaicin (100 nM) and NADA (100 nM) were determined. In control and capsaicin-desensitized animals histamine at 10 μM was applied onto the dura mater for 3 min after completion of the measurement of the vanilloid-induced blood flow changes. To study the role of CB1 receptor activation on endovanilloid-induced meningeal vasodilatation, the effects of anandamide (10 μM) administrations were tested before and after CB1 receptor antagonist AM 251 (100 μM) application for 5 min. In some experiments the vasodilatory effect of anandamide (10 μM) was tested also after blocking both CB1- and CGRP receptors with pre-treatment of the dura mater with AM 251 (100 μM) and CGRP_8–37_ (100 μM).

Anandamide, NADA and AM 251 were purchased from Tocris Bioscience (United Kingdom), all the other drugs from Sigma-Aldrich Chemie Gmbh (Germany).

### Measurement of dural blood flow and evaluation of data

Meningeal blood flow was recorded on-line with a needle type probe of a laser Doppler flowmeter (Perimed, Sweden) over branches of the middle meningeal artery. To minimize flow signals from the cortical blood vessels, recording sites lying distant from the visible cortical blood vessels were chosen. Under these circumstances, changes in the laser Doppler signal almost exclusively reflected the changes in meningeal blood flow [35]. Meningeal blood flow (measured in perfusion units, PU), systemic blood pressure and body temperature were stored and processed with the Perisoft program (Perimed, Sweden). The basal blood flow was the mean flow value measured during a 5-min period prior to drug application. The percentage changes induced in the blood flow by topical applications of capsaicin, endovanilloids and histamine were determined as mean flow values relative to the basal blood flow, calculated as average for the 3 min application periode. The effects of TRPV1-, CGRP- and CB1-receptor antagonists on the endovanilloid-induced blood flow changes were determined by comparing the changes in blood flow in response to NADA before and after the application of the respective antagonist(s).

### Measurement of meningeal CGRP release in vitro

The release of CGRP from the dural afferent nerves was measured by the method of Ebersberger et al. [37]. Control rats were deeply anaesthetized with thiopental sodium (150 mg/kg, i.p.) and decapitated. After removal of the skin and muscles, the skull was divided into halves along the midline and the cerebral hemispheres were removed. The skull preparations were washed with SIF at room temperature for 30 min and then mounted in a humid chamber at 37 °C. The cranial fossae were filled with 300 μl of SIF solution. In one series of experiments three consecutive samples of the superfusate were collected at periods of 5 min by carefully removing the content of the skull halves with a pipette. The first sample served as reference indicating the basal CGRP release in the dura mater. The second sample was collected after incubation in the presence of NADA at 100 nM and the third sample after capsaicin at 100 nM. In another series of experiments the effect of CB1 receptor antagonist pre-treatment was studied on the anandamide-(10 μM) induced CGRP release; in these preparations after measuring the basal CGRP release, anandamide (10 μM) was applied twice for five minutes. CB1 receptor antagonist AM 251 was applied at 100 μM prior to the second anandamide application. All samples were frozen at −70 °C for later analysis.

The CGRP contents of the samples were measured by enzyme-linked immunoassay kit (Bertin Pharma, France). The absorbance of the reaction product representing the CGRP content of the sample was determined photometrically, using a microplate reader (DYNEX MRX). CGRP content was measured in pg/ml, changes induced in CGRP release by vanilloids were expressed as percentage changes relative to the basal release calculated for the 5-min application period. Changes in anandamide-induced CGRP release were compared before and after blocking the CB1 receptors with its antagonist.

### Statistics

All values were expressed as mean ± SEM. Statistical analysis of the data was performed using Statistica 12 (StatSoft, Tulsa, OK, USA). For the statistical comparisons one-way ANOVA followed by the Bonferroni test was used. A probability level of *p* < 0.05 was regarded as statistically significant.

## Results

### Effect of topical application of vanilloids on dural blood flow

In accord with previous findings [9], in control rats topical application of the exogenous vanilloid capsaicin at a concentration of 100 nM produced significant increases in blood flow which amounted to 16.4 ± 3.4 % (*n* = 7, *p* = 0.002) of the baseline value. Administration of the endovanilloid anandamide resulted in minor changes of meningeal blood flow. At concentrations of 100 nM and 1 μM anandamide slightly increased meningeal blood flow by 3.4 ± 1.5 (*n* = 8, *p* = 0.06) and 2.5 ± 1 % (*n* = 10, *p* = 0.033), respectively, whereas at the highest concentration tested in our experiments (10 μM), it had a slight vasoconstrictor effect decreasing blood flow by 2.1 ± 0.8 % (*n* = 10, *p* = 0.022). Topical application of NADA induced a significant dose-dependent increase in meningeal blood flow which amounted to 7.4 ± 2 % (*n* = 10, *p* = 0.003) and 24 ± 4.7 % (*n* = 11, *p* = 0.016) at concentrations of 10 nM and 100 nM, respectively. However, NADA applied at 1 μM decreased meningeal blood flow by 7.7 ± 4.3 % (*n* = 6, *p* = 0.09; Table 1). Systemic blood pressure was not influenced by topical applications of endovanilloids or capsaicin. Systolic blood pressure measured before and after the applications of the compounds was 110 ± 22.4 and 92 ± 27.8 mmHg (*p* = 0.20).

**Table 1:** Effects of topical applications of anandamide and NADA on meningeal blood flow

	Concentration	Changes in blood flow (%)	Number	*p*-value
Anandamide	100 nM	3.4 ± 1.5	8	0.06
	1 μM	2.5 ± 1^*^	10	0.033
	10 μM	−2.1 ± 0.8^*^	10	0.022
NADA	10 nM	7.4 ± 2^*^	10	0.003
	100 nM	24 ± 4.7^*^	11	0.016
	1 μM	−7.7 ± 4.3	6	0.09

^*^: Statistically different from the baseline (*p* < 0.05)

### Effect of systemic capsaicin desensitization on vanilloid-induced changes in meningeal blood flow

**Fig. 1:**
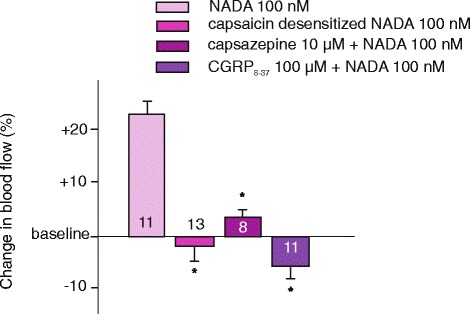
Effect of systemic capsaicin desensitization and preapplication of capsazepine or CGRP_8–37_ on NADA-induced changes in meningeal blood flow. Percentage changes in blood flow were calculated as mean ± SEM for the 3-min application period relative to the baseline. The number of experiments is indicated in the bars. *: statistically different from the corresponding control values (*p* < 0.05)

In accord with our previous observations, in capsaicin-desensitized animals the vasodilatory effect of capsaicin (100 nM) was completely abolished. It was 99.8 ± 1 % of the basal flow (*n* = 10, *p* = 0.91). Similarly, in capsaicin-desensitized animals the administration of NADA (100 nM) resulted in a slight decrease (0.9 ± 1.2 %, *n* = 13, *p* = 0.018) in blood flow instead of the marked vasodilatory effect observed in control animals (Fig. 1). Systolic blood pressure of capsaicin-desensitized animals was in the same range as in controls (97 ± 17.3 mmHg) and it was not affected by the applications of the compounds. Desensitization with capsaicin did not interfere with local vascular mechanisms involved in vasodilatation, since topical application of histamine resulted in similar increases of 22.3 ± 0.7 (*n* = 10) and 19.3 ± 0.5 % (*n* = 7, *p* = 0.64) in meningeal blood flow in control and desensitized rats, respectively.

### Effect of capsazepine and CGRP_8–37_ on changes of meningeal blood flow elicited by endovanilloids

To obtain pharmacological evidence for the involvement of TRPV1 receptor activation and consequent CGRP release in endovanilloid-induced meningeal vasodilatation, the specific TRPV1 receptor antagonist capsazepine (10 μM) or the CGRP receptor antagonist CGRP_8–37_ (100 μM) were applied topically prior to NADA (100 nM). Administrations of capsazepine and CGRP_8–37_ did not significantly influence basal blood flow, but resulted in a significant inhibition of the vasodilatory effect of NADA. Following the application of capsazepine the blood flow increasing effect of NADA was only 2.23 ± 3.3 % (*n* = 8, *p* = 0.019), while after the application of CGRP_8–37_, NADA elicited a moderate decrease in meningeal blood flow by 4.82 ± 1.42 % (*n* = 11, *p* < 0.001; Fig. 1).

### Modulating effect of CB1 receptor activation on endovanilloid-induced changes in meningeal blood flow

**Fig. 2:**
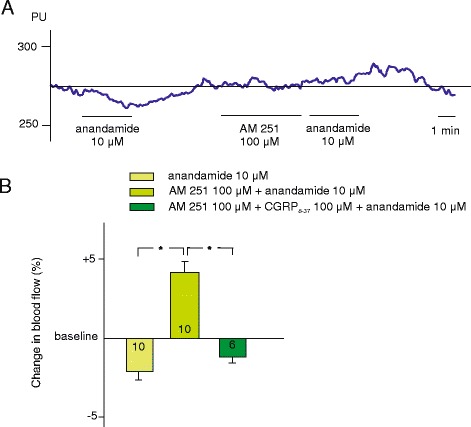
Effect of AM 251 and CGRP_8–37_ on anandamide-induced vasodilatation in the dura mater. Original recording (**a**) and statistical evaluation (**b**) of changes induced by anandamide (10 μM) before and after the application of AM 251 (100 μM) or AM 251 (100 μM) and CGRP_8–37_ (100 μM). Percentage changes in blood flow were calculated as mean ± SEM for the 3-min application period relative to the baseline. The number of experiments is indicated in the bars. *: statistically different from the effect of anandamide after AM 251 pretreatment (*p* < 0.05)

In control rats, dural application of a CB1 receptor antagonist AM 251 (100 μM) did not affect basal meningeal blood flow. Although anandamide, at a concentration of 10 μM had a moderate vasoconstrictor effect on meningeal blood vessels, following the administration of AM 251 prior to the application of anandamide, a moderate but significant increase in meningeal blood flow was recorded. Indeed, the slight decrease by 2.1 ± 0.8 % produced by anandamide (10 μM) turned into an increase by 4.1 ± 0.6 % after pre-treatment with the CB1 antagonist (*n* = 10, *p* < 0.001). This vasodilatory effect of anandamide was abolished after additional blocking of CGRP receptors by pre-treatment with CGRP_8–37_ (100 μM) resulting in a decrease of blood flow by 1.1 ± 1.8 % (*n* = 6, *p* = 0.005; Fig. 2).

### Effect of endovanilloids on the release of CGRP in the in vitro dura mater preparation

**Fig. 3:**
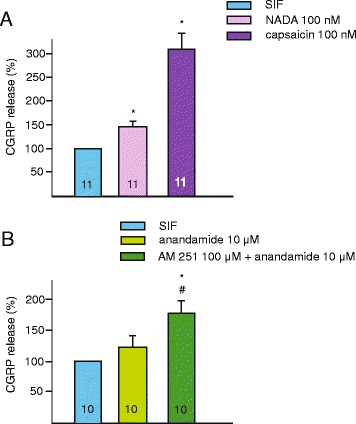
Effects of endovanilloids on meningeal CGRP release. Relative changes in the CGRP concentration are indicated after NADA (100 nM) and capsaicin (100 nM) applications (**a**) and after anandamide (10 μM) application with and without prior AM 251 (100 μM) treatment (**b**). The number of experiments is indicated in the bars. *: statistically different from the CGRP releasing effect of SIF, #: statistically different from the CGRP releasing effect of anandamide (*p* < 0.05)

The basal release of CGRP amounted to 22.6 ± 5 pg/ml in the in vitro dura mater preparations. NADA at 100 nM induced a marked increase in the release of CGRP amounting to 140.3 ± 16.2 % of the basal release (*n* = 11, *p* = 0.024). In this series of experiments the capsaicin-induced CGRP release was also measured following the challenge with NADA in the same preparation. The capsaicin-induced release of CGRP amounted to 328.8 ± 63.6 % of the basal value (*n* = 11, *p* = 0.008). Under these experimental conditions, the capsaicin-induced peptide release was taken as an indication of the functional integrity of the dura mater preparations. In the other series of experiments, the anandamide (10 μM)-induced release of CGRP was studied. Anandamide-induced release of CGRP amounted to 122.2 ± 9.6 % (*n* = 10, *p* = 0.08) of the basal, while after blocking the CB1 receptors with AM 251 (100 μM) an increase of 170.4 ± 23.7 % (*n* = 10) was measured. The changes in anandamide-induced peptide release measured after blocking the CB1 receptors were significantly different both from the baseline release and the anandamide-induced CGRP release (SIF vs. anadamide + AM 251: *p* = 0.05; anandamide vs. anadamide + AM 251: *p* = 0.04; Fig. 3).

## Discussion

The present findings furnish evidence for the vascular actions of anandamide and NADA in the dura mater encephali and suggest that endovanilloid compounds may bear significance in the activation of the trigeminal nocisensor complex implicated in the pathophysiology of headaches [3, 9, 19, 38]. The results indicate, that endogenous membrane lipid metabolites which have been already identified in primary sensory ganglion cells may activate trigeminal chemosensitive afferent nerves which express the TRPV1 nociceptor ion channel. Available experimental evidence indicates that activation of chemosensitive primary sensory neurons plays an important role in the pathophysiology of certain headaches. For example, it has been demonstrated that, in a rat experimental headache model, activation of meningeal TRPV1 receptors by capsaicin resulted in the release of CGRP from trigeminal afferent nerves. Further, release of CGRP and expression of c-fos in the trigeminal nucleus caudalis were significantly inhibited by TRPV1 antagonists [14]. The release of vasoactive neuropeptides, in particular CGRP from both central and peripheral trigeminal terminals modulates the activity of second order neurons of the trigeminal nucleus caudalis [39] and increases meningeal blood flow [9, 35]. Hence, measurements of the changes in dural blood flow in vivo, and the release of CGRP from meningeal sensory nerves in vitro, are reliable indicators of the activation of trigeminal nociceptive primary sensory neurons [9, 40–42]. Endovanilloids are defined as endogenous ligands of the TRPV1 receptor and are synthetized or may be taken up by sensory ganglion neurons [30, 43]. Anandamide and NADA are two membrane lipid metabolites present in primary sensory ganglion neurons acting on both TRPV1 and CB1 receptors with different efficacies. The cellular concentration of endovanilloids may be elevated either by increased activity of the synthetizing enzymes and/or by increased endovanilloid transport across the cell membrane [44]. Several lines of evidence suggest that anandamide binds to the same binding site of the TRPV1 receptor as capsaicin [45]. Further, anandamide may activate the TRPV1 receptors under experimental and pathophysiological conditions leading to sensitization of nociceptive primary afferent neurons [46–48]. Although tissue content of anandamide measured under physiological conditions is moderate [49], its level may increase through neurogenic inflammatory processes mediated by meningeal peptidergic nociceptive afferents and mast cells [19].

The present findings indicate that the vasoregulatory propensities of anandamide and NADA are different in the trigeminovascular system. While anandamide induced only slight, if any, increase, NADA produced a marked increase in meningeal blood flow. This difference may be explained by the different activity of these agents on TRPV1 and CB1 receptors [50]. The application of endovanilloids at higher concentrations turned the vasodilatory effects of lower concentrations of anandamide and NADA into slight vasoconstriction. This phenomenon is similar to the concentration-dependent effects of the archetypal exogenous vanilloid capsaicin on mesenteric, renal and meningeal blood flow [9, 51–53]. Capsaicin-induced vasoconstriction is generally regarded as a direct vascular action of capsaicin [54–56], although a TRPV1 receptor mediated effect can not be fully excluded [57]. Systemic pretreatment of the animals with capsaicin producing profound depletions of sensory neuropeptides CGRP and SP from afferent nerves markedly inhibited the vasodilatory effect of endovanilloids. In contrast, histamine-induced vasodilatation mediated by a direct action on endothelial and smooth muscle receptors was not affected by capsaicin-desensitization [10, 58]. Capsazepine, a specific antagonist of the TRPV1 receptors and blockage of CGRP receptors by CGRP_8–37_ inhibited the NADA-induced vasodilatation. The present findings therefore suggest that, besides exogenous vanilloids, endovanilloids can also elicit sensory neurogenic meningeal vasodilatation involving chemosensitive afferent nerves which express the TRPV1 receptor.

The moderate vasodilatory effect of anandamide in our in vivo blood flow model can be explained by its dual effects on both TRPV1 and presynaptic CB1 receptors, the latter inhibiting the release of CGRP from trigeminal nerve fibres [59]. Immunhistochemical studies revealed the presence of CB1 receptors on trigeminal nerve fibres and ganglion cells [59, 60]. The relatively strong activity of anandamide on CB1 receptors may interfere with its endovanilloid action stimulating TRPV1 receptors and CGRP release in the meningeal tissue. Our notion about the interaction between TRPV1 and CB1 receptor activation in the meningeal blood flow regulatory effect of anandamide application was supported by the observation, that blockage of CB1 receptors with AM251 has a potentiating effect on anandamide-induced vasodilatation. This vasodilatation was induced by the enhanced release of the sensory vasodilator neuropeptide CGRP, since additional blockage of CGRP receptors abolished the anandamide-induced vasodilatation after CB1 receptor antagonism. Although in vivo anandamide failed to significantly increase meningeal blood flow, in vitro a slight but non-significant increase in meningeal CGRP-release was measured after the application of anandamide at the same concentration (10 μM). The putative blood flow increasing effect of the moderate amount of CGRP released by anandamide is probably counterbalanced by its direct vasoconstrictor effect. Blocking the presynaptic CB1 receptors induced a more than threefold increase in the amount of CGRP released in the in vitro dura mater preparation; the vasodilator effect of this higher amount of CGRP could override the direct vasoconstrictor effect of anandamide. After CB1 receptor blockade, the anandamide-induced vasodilatation was mediated entirely by CGRP, since it was completely abolished by pretreatment with the CGRP antagonist.

## Conclusions

In conclusion, the present study demonstrated that similar to exogenous vanilloid compounds, endovanilloids synthesized or taken up by sensory neurons are capable of the activation of trigeminovascular nociceptive afferent nerves resulting in the release of CGRP and a consequent increase in meningeal blood flow. Chemosensitive afferents expressing the TRPV1 receptor may contribute significantly not only to the vascular reactions but also to the nociceptive mechanisms of the dura mater possibly associated with the pathomechanisms of headaches. Increased production and/or uptake of endovanilloids may be implicated in the sustained activation of the trigeminal sensory system leading to peripheral and/or central sensitization of the nociceptive pathway and, eventually head pain.

## Abbreviations

Andamide: arachidonylethanolamide
CB: cannabinoid
CGRP: calcitonin gene-related peptide
NADA: N-arachidonoyl-dopamine
SIF: synthetic interstitial fluid
SP: substance P
TRPV1: transient receptor potential vanilloid 1
